# Effects of Moderate-Intensity Aerobic Exercise on Clinical Symptoms and Physiological Outcomes in Young Adults with Persistent Allergic Rhinitis: A Randomized Controlled Trial

**DOI:** 10.3390/ijerph23050611

**Published:** 2026-05-05

**Authors:** Kanphatson Kerdkaew, Phisut Rattanathamma, Wannaporn Tongtako, Timothy Mickleborough, Bulin Jirapongsatorn

**Affiliations:** 1 Department of Sport and Exercise Science, School of Medicine, Walailak University, Nakhon Si Thammarat 80160, Thailand; kanphatson.ke@wu.ac.th; 2 Center of Excellence in Tropical Pathobiology, Walailak University, Nakhon Si Thammarat 80160, Thailand; 3 Walailak University Hospital, Walailak University, Nakhon Si Thammarat 80160, Thailand; phisut.ra@wu.ac.th; 4 Area of Exercise Physiology, Faculty of Sports Science, Chulalongkorn University, Bangkok 10330, Thailand; wannaporn.t@chula.ac.th; 5 Department of Kinesiology, School of Public Health, Indiana University, Bloomington, IN 47405, USA; tmickleb@iu.edu

**Keywords:** allergic rhinitis, aerobic exercise, pulmonary function, fractional exhaled nitric oxide (FeNO), symptoms

## Abstract

**Highlights:**

**Public health relevance—How does this work relate to a public health issue?**

- Persistent allergic rhinitis significantly impacts daily productivity and quality of life, necessitating accessible and scalable management strategies.
- This study addresses the need for non-pharmacological adjuncts to manage chronic respiratory symptoms and airway inflammation in the adult population.

**Public health significance—Why is this work of significance to public health?**

- An eight-week moderate-intensity aerobic exercise program substantially improves both subjective symptom severity and objective physiological markers, including Peak Nasal Inspiratory Flow (PNIF) and lung function (FEV_1_, FVC).
- The intervention demonstrates a significant reduction in eosinophilic airway inflammation (FeNO), supporting the “one airway, one disease” framework in public health management.

**Public health implications—What are the key implications or messages for practitioners, policy makers and/or researchers in public health?**

- Structured aerobic exercise is a safe, low-cost, and feasible “physical activity prescription” that can be integrated into community-based care and routine clinical practice.
- These findings empower patients toward self-management and provide an evidence-based approach to mitigate the socioeconomic burden of chronic allergic conditions.

**Abstract:**

Allergic Rhinitis (AR) is an IgE-mediated inflammatory disorder that impairs quality of life and systemic function. Following the ‘one airway, one disease’ paradigm, AR-related inflammation often extends to the lower respiratory tract. This randomized controlled trial investigated the effects of an 8-week moderate-intensity aerobic exercise (MOA) program on clinical symptoms, nasal airflow, airway inflammation, pulmonary function, and cardiorespiratory parameters in young adults with physician-confirmed persistent AR. To isolate the exercise effects, all participants discontinued antihistamines, corticosteroids, and leukotriene antagonists before and during the study period. Eighteen participants were allocated to either the MOA group (*n* = 9), which performed treadmill walking or jogging at 50–60% heart rate reserve three times per week for eight weeks, or a control group (CON, *n* = 9) that maintained usual daily activities. Clinical symptoms, peak nasal inspiratory flow, fractional exhaled nitric oxide, pulmonary function, heart rate, blood pressure, aerobic fitness, and perceived exertion were assessed at baseline, week 4, and week 8 using standardized procedures. Compared with baseline and the CON group, the exercise intervention resulted in significant reductions in nasal congestion, itching, sneezing, and rhinorrhea, accompanied by increased nasal airflow and reduced airway inflammation. Pulmonary function indices and cardiorespiratory parameters also improved following training. These findings suggest that moderate-intensity aerobic exercise may offer a valuable non-pharmacological approach to support conventional care, potentially enhancing respiratory and physiological outcomes in young adults with persistent AR.

## 1. Introduction

AR is an immunoglobulin E (IgE)-mediated inflammatory disorder of the nasal mucosa and a common chronic condition worldwide. Its core symptoms—sneezing, rhinorrhea, nasal itching, and congestion—affect daily functioning, sleep, and overall quality of life [1]. AR is also linked to broader physiological consequences, including persistent fatigue and an elevated risk of respiratory comorbidities such as asthma and sinusitis [2].

AR is characterized by chronic airway inflammation that influences both the upper and lower respiratory tracts. Pulmonary function parameters, such as Forced Vital Capacity (FVC) and Forced Expiratory Volume in one second (FEV_1_), often reflect these changes and demonstrate the interconnected nature of nasal and bronchial physiology [3]. Fractional Exhaled Nitric Oxide (FeNO) is a widely used biomarker for eosinophilic airway inflammation and is valuable for assessing disease severity under the “one airway, one disease” paradigm [4]. Age-related alterations in immune and autonomic function may further modify symptom expression and cardiovascular responses [5,6].

Pharmacological treatments, including intranasal corticosteroids and antihistamines, provide symptomatic relief but do not fully correct the underlying inflammatory dysregulation. Consequently, non-pharmacological strategies have gained attention. Systemic evidence suggests that exercise serves as a potent immunomodulatory tool; regular moderate activity induces a shift toward a Th1-type cytokine profile, effectively counterbalancing the Th2-dominant allergic response. This modulation is characterized by a reduction in pro-inflammatory markers and an enhancement of anti-inflammatory cytokines, which may stabilize the mucosal immune environment [7,8]. Specifically, moderate aerobic exercise has shown anti-inflammatory and autonomic benefits in AR and related respiratory conditions [9,10]. Exercise intensity and environmental conditions, particularly temperature, may influence nasal blood flow and symptom expression; for example, activity performed at approximately 25 °C has been shown to reduce congestion and sneezing [11,12].

Although previous studies examined acute exercise responses, evidence regarding the long-term effects of structured moderate-intensity aerobic training on key physiological markers—including PNIF, FeNO, pulmonary function, and cardiorespiratory parameters—remains limited. Furthermore, few studies have simultaneously assessed these outcomes under controlled environmental conditions, leaving a gap in understanding how chronic aerobic exercise influences both nasal and systemic physiology in persistent AR.

This study investigated the effects of an eight-week moderate aerobic exercise program on clinical symptoms and physiological outcomes in young adults with persistent AR. The primary aim was to evaluate the efficacy of the exercise intervention in reducing AR symptom severity, as assessed by the Rhinitis Symptom Score (RSS), while the secondary aims were to determine the effects of the program on objective nasal patency through Peak Nasal Inspiratory Flow (PNIF), assess changes in eosinophilic airway inflammation via Fractional Exhaled Nitric Oxide (FeNO), and evaluate improvements in pulmonary function including FVC and FEV_1_, as well as cardiorespiratory parameters such as VO_2_max, heart rate, and blood pressure. We hypothesized that exercise under controlled indoor conditions would significantly alleviate symptom severity while simultaneously enhancing both upper and lower airway function and overall systemic physiology.

## 2. Materials and Methods

### 2.1. Participants and Trial Design

Twenty young adults (18–35 years) with persistent AR were screened following public recruitment via faculty social media platforms and posters displayed at the Walailak University Hospital medical center. Interested individuals underwent a structured telephone interview before a formal diagnostic confirmation by a physician, with the study conducted between June and August 2025. Potential participants were recruited through public advertisements and screened for eligibility based on the predefined inclusion and exclusion criteria. Detailed flow of participants from recruitment to final analysis is presented in the Section 3, in accordance with the CONSORT 2025 statement (File S1).

Eligible participants were nonsmokers who had been physically inactive—defined as engaging in less than 150 min of moderate-intensity activity per week as assessed by self-reported physical activity history—for at least three months prior to enrollment [13]. Throughout the 8-week study, all participants were instructed to maintain their habitual daily routines and refrain from starting any new structured exercise outside of the assigned intervention to ensure that observed physiological changes resulted solely from the study protocol. Participants were randomly assigned to either the CON group or the MOA group using a computer-generated sequence. To maintain allocation concealment, assignments were kept in sequentially numbered, opaque, sealed envelopes, opened only after baseline assessments. There were no protocol deviations or changes to outcomes after the trial commenced.

### 2.2. Inclusion and Exclusion Criteria

Inclusion required persistent AR symptoms (occurring ≥4 days per week for ≥4 consecutive weeks) and a baseline rhinitis symptom score ≥7, consistent with EAACI and ARIA recommendations for moderate-to-severe persistent AR [14,15]. All diagnoses were physician-confirmed, supported by documented allergic sensitization in participants’ medical records. We excluded individuals with underlying respiratory, cardiovascular, or musculoskeletal disorders, as well as those using regular supplements. All participants discontinued antihistamines, corticosteroids, and leukotriene antagonists before and during the study period. Further exclusion criteria included any acute illness preventing exercise, withdrawal of consent, and clinically diagnosed respiratory conditions that could impair breathing during exercise, such as nasal polyps or chronic obstructive pulmonary disease (COPD).

Participants were recruited from the local community in Nakhon Si Thammarat, Thailand. All moderate aerobic exercise sessions and physiological assessments were performed in a controlled laboratory setting at Walailak University. Eligible participants were randomly assigned to either the CON group or the MOA group. All participants provided written informed consent before participation. The study protocol adhered to the Declaration of Helsinki and was approved by the Human Research Ethics Committee of Walailak University (WUEC-25-188-01). Additionally, the trial was prospectively registered with the Thai Clinical Trials Registry (TCTR ID: TCTR20260117002). The completed CONSORT 2025 checklist is provided as Supplementary File S1.

### 2.3. Exercise Protocol

Participants in the MOA group performed a supervised, moderate-intensity aerobic program consisting of 60-min treadmill sessions (walking or jogging), three times weekly for eight weeks. To ensure a safe transition for physically inactive participants, each session began with a 10-min warm-up, followed by 40 min of moderate-intensity aerobic exercise, and concluded with a 10-min cool-down. Exercise dosage was individually prescribed at 50–60% of heart rate reserve (HRR) using the Karvonen formula:

HR target = [HR_max − HR_rest) × %intensity] + HR_rest

All sessions took place in a thermally controlled environment (25 °C; 40–60% relative humidity), with each session supervised by a trained exercise physiologist to ensure safety and protocol adherence. Participants in the CON group were instructed to maintain their habitual daily routines and refrain from initiating any new structured exercise programs during the eight-week study period. To ensure compliance, their physical activity levels were monitored through weekly self-reported activity logs.

### 2.4. Outcome Measurements

Outcome assessments were conducted at baseline, week 4, and week 8 under standardized laboratory conditions.

General Characteristics: General characteristics were assessed to evaluate participants’ baseline physical condition. Body weight and body mass index (BMI) were assessed using a bioelectrical impedance analyzer (ACCUNIQ BC 380, Daejeon, Republic of Korea).

Clinical Symptom Variables: Clinical symptoms of AR were assessed using the Rhinitis Symptom Score (RSS) [15], following the standardization recommendations of the EAACI Position Paper, which evaluates congestion, rhinorrhea, sneezing, and nasal itching on a standardized numerical scale, with higher scores indicating greater symptom severity. They were recorded three times: baseline (0 min), week 4, and week 8. Rhinitis symptoms were evaluated using the Rhinitis Symptom Score questionnaire.

Peak Nasal Inspiratory Flow (PNIF): Nasal patency was assessed via peak nasal inspiratory flow (PNIF) using an In-Check Nasal Inspiratory Flow Meter (Clement Clarke International Ltd., Harlow, UK). Participants performed three maximal nasal inspirations following full expiration, with the highest value recorded for analysis. To minimize potential bias, the assessors remained blinded to group allocation throughout the measurement process.

Fractional Exhaled Nitric Oxide (FeNO): Airway inflammation was evaluated by measuring fractional exhaled nitric oxide (FeNO) using a calibrated NIOX VERO^®^ analyzer (Circassia Pharmaceuticals, Oxford, UK) following American Thoracic Society (ATS) [15] guidelines. Participants exhaled at a constant flow rate after full inhalation to ensure measurement consistency. The blinding of assessors was maintained during these assessments, where elevated FeNO values served as indicators of eosinophilic airway inflammation typically associated with AR.

Pulmonary function: Pulmonary function, including Forced Vital Capacity (FVC) and Forced Expiratory Volume in one second (FEV_1_), was measured using a computerized spirometer (VO_2_max Tracker Ergospirometer, MES, Kraków, Poland) following American Thoracic Society (ATS) standards. The best values from three acceptable maneuvers were recorded as absolute values (L) and percentages of predicted values (%predicted). Predicted values were calculated using the reference equations for the Thai population to account for variations in age, sex, and height, to ensure ethnic specificity and higher accuracy for our study population [13,16,17].

All physiological measurements were conducted by a single trained investigator to ensure intra-rater consistency. The instruments used are clinically validated with established high reliability: PNIF (ICC = 0.89) [18], FeNO (CV = 0.066) [19], and spirometry (ICC = 0.94). Spirometry maneuvers were performed in triplicate and evaluated based on the ATS/ERS 2019 technical standards, requiring a repeatability tolerance of ≤0.150 L between the two largest FEV_1_ and FVC values [16].

Cardiorespiratory parameters: Cardiorespiratory parameters, including Resting heart rate and blood pressure, were measured using an automated monitor (Microlife BP B1 Standard, Taipei, Taiwan) after 10 min of seated rest. Exercise tolerance was evaluated using the YMCA 3-min step test. VO_2_max was estimated using sex-specific equations from Van Kieu et al. [20]:

Males: VO_2_max = 70.597 − 0.246 × (Age) + 0.077 × (Height) − 0.222 × (Weight) − 0.147 × (HR)

Females: VO_2_max = 70.597 − 0.185 × (Age) + 0.097 × (Height) − 0.246 × (Weight) − 0.122 × (HR)

Peripheral oxygen saturation (SpO_2_): Peripheral oxygen saturation (SpO_2_) was measured using a pulse oximeter (AccuMed, Houston, TX, USA). Participants were instructed to remain still while the researcher opened the clip sensor and placed the participant’s index finger between the rubber cushions. The device automatically recorded the SpO_2_ value once a stable signal was detected.

Heart Rate Monitoring: Heart rate (HR) was continuously measured using a chest strap heart rate monitor (Polar H10, Kempele, Finland). The electrode areas of the strap were lightly moistened before placement to optimize conductivity. The strap was positioned around the participant’s chest, just below the pectoral muscles, and adjusted to ensure a secure and comfortable fit.

Rating of Perceived Exertion (RPE): Participants’ subjective exertion levels were assessed using the modified Borg CR-10 scale, ranging from 0 (rest) to 10 (maximal effort). To ensure consistent scoring, specific verbal descriptors were provided: scores of 2–3 represented light intensity, 4–5 moderate, 6–7 vigorous, and 8–9 very hard intensity. RPE was recorded immediately following a standardized submaximal 3-min step test at baseline, week 4, and week 8 to evaluate longitudinal changes in perceived effort at a constant workload.

### 2.5. Environmental Control

All assessments were conducted at the same time of day (16:00–18:00) in a controlled room (25 ± 1 °C; 40–60% relative humidity). Participants abstained from caffeine, alcohol, and strenuous exercise for 24 h before testing. Equipment—including the PNIF meter, spirometer, and FeNO analyzer—was calibrated daily.

### 2.6. Data Analysis

Statistical analyses were performed using SPSS Version 25.0 (IBM Corp., Armonk, NY, USA). The sample size was prospectively determined based on a power analysis for the primary outcome (Total Rhinitis Symptom Score; TRSS) from 8.00 to 5.00 units. Drawing from clinical outcomes reported by Tongtako et al. [21], where moderate-intensity exercise yielded a significant symptom reduction (approx. 3.0 units), we anticipated a large effect size (Cohen’s f = 0.40). A power analysis using G*Power 3.1.9.7 for a 2 × 2 mixed-model ANOVA (α = 0.05, power = 0.90) indicated that a minimum of 14 participants was required. We enrolled 18 participants to account for potential attrition.

Statistical analyses were performed using SPSS Version 25.0 (IBM Corp., Armonk, NY, USA). Data are presented as means ± standard deviation. Normality was assessed using the Shapiro–Wilk test. Independent samples *t*-tests were used to compare baseline characteristics between groups. A two-way mixed-model repeated-measures ANOVA was conducted to evaluate the effects of time, group, and their interaction across all dependent variables (HR, SpO_2_, RPE, RSS, PNIF, FeNO, pulmonary function, and cardiorespiratory parameters). Given the multiple outcomes analyzed, post hoc comparisons were conducted using the Bonferroni correction to mitigate the risk of Type I error and maintain statistical stringency. Effect sizes were calculated as partial eta squared ( $n_{p}^{2}$ ), and the threshold for statistical significance was set at *p* < 0.05.

## 3. Results

**Figure 1:**
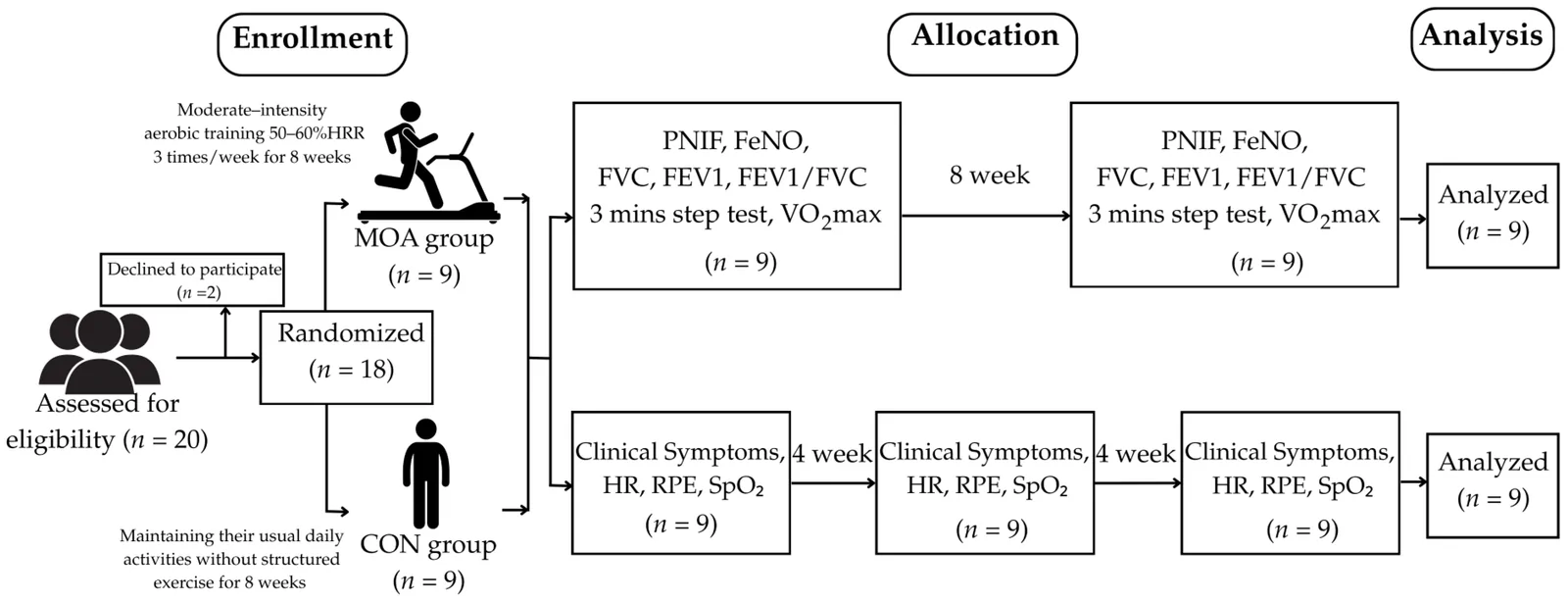
Flow diagram of participant allocation and analysis.

There were 18 participants in the research collection (Figure 1). The general characteristics of the participants are presented in Table 1.

### 3.1. Participant Flow and Recruitment

A total of 20 young adults with persistent AR were initially screened for eligibility between June and August 2025. Of these, 2 participants were excluded for not meeting the inclusion criteria (*n* = 1 for irregular symptoms; *n* = 1 for engaging in regular exercise). The remaining 18 participants were enrolled and randomly allocated to either the MOA group (*n* = 9) or the CON group (*n* = 9). All 18 participants completed the full 8-week protocol and were included in the final analysis. No adverse events related to the exercise intervention were reported.

### 3.2. Baseline Characteristics of Participants

Baseline demographic and clinical characteristics were comparable between the CON group and MOA groups, as summarized in Table 1. The study population (*n* = 18) had a mean age of 22.44 ± 4.5 years, with no significant differences observed between groups for age, weight, height, or BMI (*p* > 0.05). Similarly, baseline RSS for congestion, itching, sneezing, and rhinorrhea showed no statistical variation (*p* > 0.05).

### 3.3. Body Weight and BMI

The anthropometric characteristics of the participants remained stable throughout the study. The two-way mixed-model ANOVA revealed no significant time × group interaction effects for both body weight and BMI. At the post-test, no significant differences were observed between the MOA and CON groups in body weight or height. These results are presented in Table 2.

### 3.4. HR, RPE, SpO_2_

Significant time × group interactions were observed for resting HR and RPE (*p* < 0.05). By week 4, the MOA group already exhibited a significantly lower resting HR (MD = −9.67; 95% CI: −17.16, −2.17; *p* = 0.015) and lower RPE (MD = −0.89; 95% CI: −1.66, −0.12; *p* = 0.027) than the CON group. These differences persisted at week 8, with the MOA group maintaining a lower HR (MD = −8.33 bpm; 95% CI: −15.61, −1.06; *p* = 0.027) and RPE (MD = −2.33; 95% CI: −3.09, −1.58; *p* < 0.001) relative to CON.

**Figure 2:**
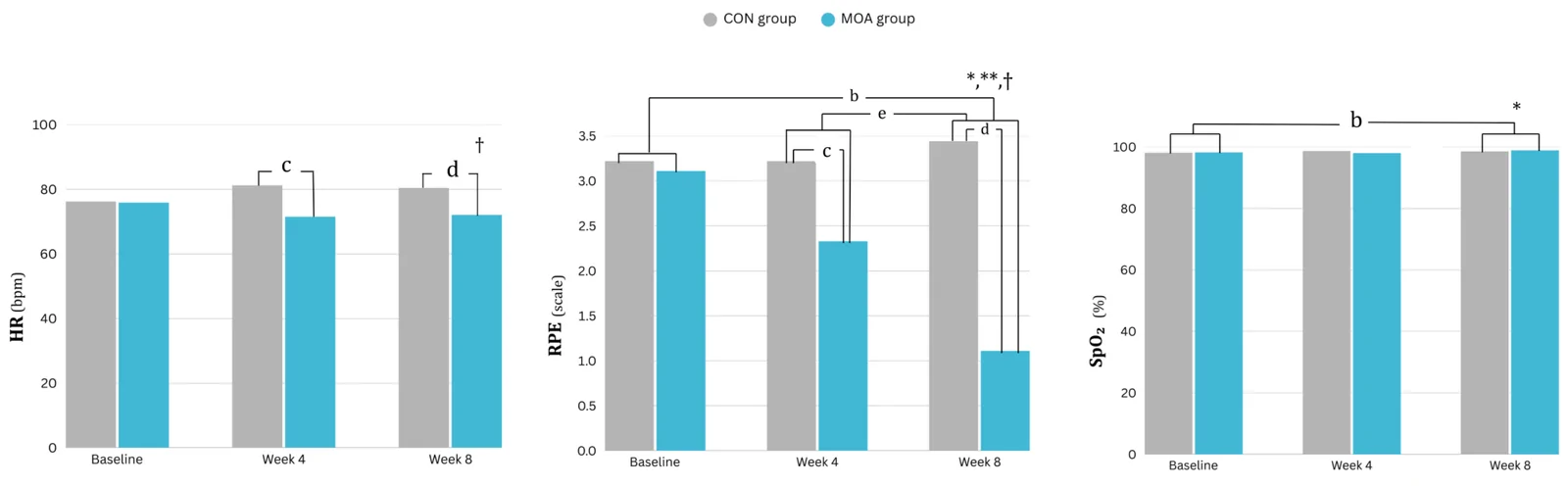
The results of the two-way repeated measures ANOVA for comparing the HR, RPE and SpO_2_ variables among baseline (Week 0), Week 4, Week 8, and between each group. Data are presented as means ± SD. * *p* < 0.05, Time, ** *p* < 0.05, Group, † *p* < 0.05, Time *×* Group, ^b^ *p* < 0.05, vs. baseline—week 8, ^e^ *p* < 0.05, vs. week 4–week 8, ^c^ *p* < 0.05, vs. group at week 4, ^d^ *p* < 0.05 vs. group at week 8. (HR = Heart rate, RPE = Rate perceived exertion, SpO_2_ = Peripheral oxygen saturation).

Regarding SpO_2_, no significant time × group interaction was observed (*p* = 0.665), nor were inter-group differences detected at any time point (all *p* > 0.05). However, a significant main effect of time (*p* = 0.042) showed that SpO_2_ increased overall from baseline to week 8 (MD = 0.56; 95% CI: 0.02, 1.09; *p* = 0.042). These results are illustrated in Figure 2 and Supplementary Figure S1.

### 3.5. Clinical Symptom Variables

**Figure 3:**
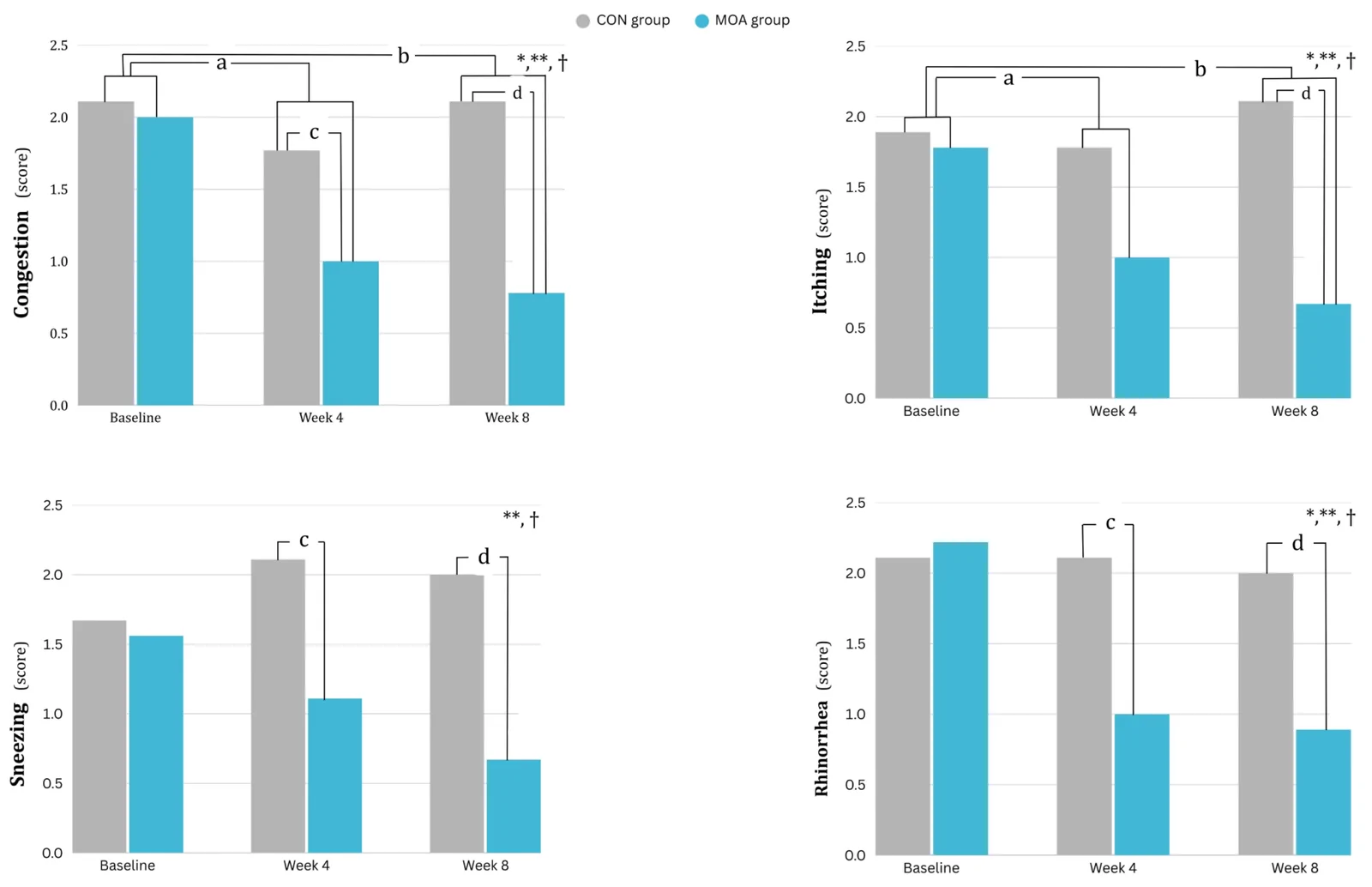
The results of the two-way repeated measures ANOVA for comparing the RSS variables among baseline (Week 0), Week 4, Week 8, and between each group. Data are presented as means ± SD. * *p* < 0.05, Time, ** *p* < 0.05, Group, † *p* < 0.05, Time × Group, ^a^ *p* < 0.05, vs. baseline—week 4, ^b^ *p* < 0.05, vs. baseline—week 8, ^c^ *p* < 0.05 vs. group at week 4. ^d^ *p* < 0.05 vs. group at week 8.

Significant time × group interactions were observed for all RSS components (*p* < 0.05). By week 4, the MOA group exhibited significantly lower scores than the CON group in congestion, sneezing, and rhinorrhea (all *p* < 0.05; MD range: 0.78–1.11). By week 8, these improvements further intensified, with the MOA group showing superior outcomes across all symptoms. Specifically, itching (MD = 1.44; 95% CI: 0.79, 2.10) and sneezing (MD = 1.33; 95% CI: 0.72, 1.95) showed the most significant reductions (both *p* < 0.001), followed by congestion (MD = 1.33; 95% CI: 0.45, 2.22; *p* = 0.005) and rhinorrhea (MD = 1.11; 95% CI: 0.46, 1.77; *p* = 0.002). Conversely, the CON group showed no significant symptomatic changes. Results are visualized in Figure 3 and Supplementary Figure S2.

### 3.6. PNIF, FeNO

Significant time × group interactions were observed for both PNIF and FeNO (*p* < 0.001). At week 8, the MOA group demonstrated marked improvements in nasal patency, with PNIF increasing significantly compared to the CON group (*p* = 0.015). This was accompanied by a substantial reduction in airway inflammation, as evidenced by lower FeNO levels in the MOA group relative to the CON group (*p* < 0.001). These results suggest that 8 weeks of moderate aerobic exercise effectively enhances nasal airflow while attenuating underlying inflammatory processes. Statistical details are summarized in Table 3 and Supplementary Figure S3.

### 3.7. Pulmonary Function

Significant time × group interactions were observed for FEV_1_%predicted (*p* = 0.001) and FVC %predicted (*p* = 0.002). Post hoc comparisons indicated that the MOA group achieved a significant increase in FVC %predicted compared to the CON group at post-test (*p* = 0.008). Although FEV_1_%predicted improved within the MOA (*p* < 0.05), between-group differences at post-test were not significant (*p* = 0.148). No significant interactions or group differences were found for the FEV_1_/FVC %predicted. Comprehensive data are presented in Table 4 and Supplementary Figure S4.

### 3.8. Cardiorespiratory Parameters

Significant time × group interactions were observed for SBP, 3-min step test performance, submaximal HR, and estimated VO_2_max (*p* < 0.05). At week 8, the MOA group showed superior cardiovascular adaptations over the CON group, marked by significant reductions in SBP (*p* = 0.011) and submaximal HR (*p* = 0.005). Aerobic capacity also improved significantly in the MOA group, with higher 3-min step test scores (*p* = 0.021) and VO_2_max levels (*p* = 0.039) compared to the CON group. Conversely, DBP showed no significant inter-group difference (*p* = 1.000). Detailed outcomes are presented in Table 5 and Supplementary Figure S5.

## 4. Discussion

The present study demonstrated that an eight-week moderate-intensity aerobic exercise program substantially improved symptom severity and multiple physiological outcomes in young adults with persistent AR. While previous research primarily focused on subjective symptom scores or isolated nasal parameters, this study advanced existing knowledge by providing a multi-dimensional assessment across the respiratory continuum. These findings reinforced that structured aerobic exercise served as an effective non-pharmacological adjunct to conventional therapies by enhancing nasal airflow, pulmonary function, and airway inflammation profiles, thereby supporting the management of chronic nasal hypersensitivity.

The increase in PNIF indicates enhanced nasal patency and is consistent with earlier work showing that moderate exercise reduces nasal resistance and congestion [22]. Prior studies have linked these changes to sympathetic activation and reduced mucosal blood flow [12,23]. Similar short-term effects have been reported in healthy adults [24]. Conducting exercise sessions under thermally controlled conditions (25 °C) helped minimize environmental influences, which is relevant because temperature and allergens can affect nasal airflow [12].

Lower FeNO values in the MOA group suggest decreased eosinophilic airway inflammation, aligning with evidence that physical activity can modulate inflammatory responses within the “one airway, one disease” framework [4,20,25]. Prior studies in asthma populations demonstrate comparable reductions in FeNO following structured exercise [26,27,28], supporting the possibility that aerobic exercise influences shared inflammatory pathways across allergic airway disorders.

The significant increases in FEV_1_, FVC, and the FEV_1_/FVC ratio in the MOA group suggest an enhancement in expiratory flow dynamics and functional lung capacity following the intervention. While exercise training does not typically alter the structural properties of lung parenchyma in healthy adults, it is well-established that aerobic training can improve the strength and endurance of the respiratory muscles [3]. In participants with AR, such training may lead to more efficient thoracic expansion and forceful expiration. Furthermore, the reduction in airway inflammation (evidenced by our FeNO results) may have contributed to decreased airway resistance, further facilitating these improvements in ventilatory parameters in people with persistent AR [21,26,29].

The 8-week intervention yielded significant cardiorespiratory adaptations, evidenced by increased estimated VO_2_max, lower resting and submaximal heart rates, and improved step-test performance. These changes collectively point toward enhanced stroke volume, peripheral oxygen reviewers’ extraction, and exercise tolerance. Interestingly, body mass remained stable across both groups throughout the study period. This suggests that the observed physiological improvements were a direct result of aerobic conditioning rather than changes in body composition or weight loss. While relative estimated VO_2_max (mL·kg^−1^·min^−1^) showed significant gains, the absolute values (L/min) followed a consistent upward trend. Although we acknowledge that submaximal estimation may yield higher absolute values compared to direct gas analysis, the robust decrease in heart rate response at a fixed workload further confirms a genuine enhancement in cardiorespiratory efficiency [30,31].

However, we acknowledge that the indirect estimation of VO_2_max carries an inherent technical variation and potential margin of error. To provide a more robust validation of the training effect, we prioritized the observed reduction in heart rate response at a fixed submaximal workload as a more stable indicator of cardiovascular efficiency. Furthermore, to maintain scientific rigor and reflect the realistic precision of these estimations, VO_2_max values have been rounded to the nearest whole number, moving away from overly specific decimal reporting [30,32,33]. Furthermore, conducting the program under standardized temperatures helped mitigate variability associated with environmental stressors [34,35]. While no adverse events were reported, suggesting the safety of the protocol, future studies are needed to formally evaluate the long-term feasibility of integrating such programs into community-based care [21,22].

The findings of this study had significant implications for public health and the practical management of AR, demonstrating that moderate-intensity aerobic exercise functioned as a feasible, scalable, and accessible health promotion strategy. Due to its low cost and minimal equipment requirements, this intervention could be integrated into community-based programs or routine care as a ‘physical activity prescription,’ empowering patients toward self-management. While previous research primarily utilized subjective scores, this multi-dimensional assessment confirmed that exercise improved both physiological capacity and symptom severity across the respiratory continuum. Importantly, the program was well-tolerated with no adverse events, providing robust evidence base for a safe non-pharmacological approach that complements standard medical therapies [21,22,33,36]. By promoting such accessible lifestyle modifications, these interventions may mitigate the socioeconomic burden of AR while enhancing the overall quality of life for individuals with chronic allergic conditions.

This study has several limitations. First, the modest sample size and eight-week duration suggest that findings should be viewed as exploratory. Second, the recruitment of young adults aged 18–35 via public announcements may introduce selection bias, potentially limiting generalizability. Regarding measurement accuracy, VO_2_max was estimated using predictive equations rather than direct gas exchange analysis, which may affect the precision of cardiorespiratory data. Furthermore, while a specific pilot reliability study was not conducted within this cohort, the high internal consistency of our raw data and its alignment with established clinical validation standards support the overall integrity of the measurements. Lastly, the absence of molecular biomarkers precludes a deeper mechanistic understanding of the observed changes. Future research should utilize larger, more diverse cohorts and employ direct metabolic measurements with longitudinal follow-ups to further refine exercise prescriptions for AR management.

## 5. Conclusions

This exploratory study indicates that eight weeks of moderate-intensity aerobic exercise may alleviate symptoms of persistent AR and improve key physiological outcomes, including nasal airflow, airway inflammation, pulmonary function, and cardiorespiratory capacity. The improvements in PNIF, FeNO, FEV_1_, FVC, and VO_2_max suggest that regular aerobic exercise can support both upper and lower airway function. These preliminary results highlight moderate-intensity aerobic exercise as a feasible and safe non-pharmacological strategy that serves as a supportive health promotion tool to complement standard treatments. Further research with larger cohorts and longer follow-up is necessary to clarify underlying mechanisms and optimize exercise recommendations for broader clinical and community use.

## Figures and Tables

**Table 1:** Baseline of Characteristics of Participants.

Variable	CON Group (*n* = 9)	MOA Group (*n* = 9)	*p* Value	t
Demographics				
Sex (Male/Female)	4/5	4/5		
Age (year)	23.22 ± 5.26	21.67± 3.71	0.480	0.73
Weight (kg)	61.94 ± 11.75	61.90 ± 19.64	0.995	0.06
Height (cm)	162.11 ± 8.58	162.78 ± 10.49	0.884	−0.15
BMI (kg/m^2^)	23.44 ± 3.15	22.81 ± 4.48	0.736	0.34
RSS				
Congestion (score)	2.11 ± 0.60	2.00 ± 0.87	0.756	0.32
Itching (score)	1.89. ± 0.78	1.78 ± 0.83	0.774	0.29
Sneezing (score)	1.67. ± 0.78	1.56 ± 0.88	0.772	0.30
Rhinorrhea (score)	2.11. ± 0.78	2.22 ± 0.67	0.750	−0.32

Data are presented as means ± SD. (BMI = Body mass index, RSS = Rhinitis symptom score).

**Table 2:** The results of the two-way mixed-model repeated-measures ANOVA for comparing the body weight and BMI among pretest and post-test between each group.

Variables	CON Group (*n* = 9)		MOA Group (*n* = 9)		*p*-Value vs. Time × Group ( $n_{p}^{2}$ )			Between-Group MD (at Week 8) [95% CI]
	Pretest	Post-Test	Pretest	Post-Test	Time	Group	Time × Group	
Body Weight (kg)	61.94 ± 11.79	63.97 ± 13.10	61.90 ± 19.64	58.96 ± 17.77	0.082 (0.01)	0.734 (0.01)	0.142 (0.13)	5.01 [−10.59, 20.61]
*p*-value vs. group ( $n_{p}^{2}$ )			0.995 (0.00)	0.506 (0.03)				
BMI (kg/m^2^)	23.43 ± 3.15	24.14 ± 3.25	22.81 ± 4.48	21.79 ± 3.97	0.773 (0.14)	0.391 (0.05)	0.129 (0.01)	2.35 [−1.28, 5.97]
*p*-value vs. group ( $n_{p}^{2}$ )			0.736 (0.01)	0.189 (0.11)				

Data are presented as means ± SD. (MD = the mean difference between the MOA and CON groups at post-test, 95% CI = 95% confidence interval of the difference, BMI = Body mass index).

**Table 3:** The results of the two-way mixed-model repeated-measures ANOVA for comparing the PNIF and FeNO among pretest and post-test between each group.

Variables	CON Group (*n* = 9)		MOA Group (*n* = 9)		*p*-Value vs. Time × Group ( $n_{p}^{2}$ )			Between-Group MD (at Week 8) [95% CI]
	Pretest	Post-Test	Pretest	Post-Test	Time	Group	Time × Group	
PNIF (L/min)	89.00 ± 22.42	82.00 ± 18.63	91.11 ± 19.17	107.33 ± 20.74 *	0.059 (0.21)	0.159 (0.12)	<0.001 (0.62)	−25.33 [−45.63, −5.03]
*p*-value vs. group ( $n_{p}^{2}$ )			0.833 (0.00)	0.015 (0.32)				
FeNO (ppb)	15.11 ± 1.36	15.66 ± 2.00	15.00 ± 0.71	10.00 ± 1.00 *	<0.001 (0.80)	<0.001 (0.61)	<0.001 (0.86)	5.63 [4.09, 7.25]
*p*-value vs. group ( $n_{p}^{2}$ )			0.831 (0.00)	<0.001 (0.78)				

Data are presented as means ± SD. * *p* < 0.05, vs. pretest within the same group. (MD = the mean difference between the MOA and CON groups at post-test, 95% CI = 95% confidence interval of the difference, PNIF = Peak Nasal Inspiratory Flow, FeNO = Fractional exhaled nitric oxide).

**Table 4:** The results of the two-way mixed-model repeated-measures ANOVA for comparing the percentage predicted values of FEV_1_, FVC, and FEV_1_/FVC among pretest and post-test between each group.

Variables	CON Group (*n* = 9)		MOA Group (*n* = 9)		*p*-Value vs. Time × Group ( $n_{p}^{2}$ )			Between-Group MD (at Week 8) [95% CI]
	Pretest	Post-Test	Pretest	Post-Test	Time	Group	Time × Group	
FEV_1_ (%predicted)	107.15 ± 24.35	102.71 ± 20.86	101.18 ± 24.07	117.58 ± 20.61 *	0.040 (0.24)	0.671 (0.01)	0.001 (0.49)	−14.87 [−35.59, 5.86]
*p*-value vs. group ( $n_{p}^{2}$ )			0.608 (0.02)	0.148 (0.13)				
FVC (% predicted)	84.44 ± 17.03	82.09 ± 15.47	91.63 ± 21.30	109.88 ± 22.76 *	0.012 (0.33)	0.061 (0.20)	0.002 (0.46)	−27.79 [−47.24, −8.34]
*p*-value vs. group ( $n_{p}^{2}$ )			0.441 (0.04)	0.008 (0.36)				
FEV_1_/FVC (%predicted)	92.91 ± 11.85	92.58 ± 13.14	91.48 ± 8.46	98.16 ± 9.36	0.112 (0.15)	0.669 (0.01)	0.082 (0.17)	−5.58 [−16.98, 5.83]
*p*-value vs. group ( $n_{p}^{2}$ )			0.773 (0.01)	0.315 (0.06)				

Data are presented as means ± SD. * *p* < 0.05, vs. pretest within the same group. (MD = the mean difference between the MOA and CON groups at post-test, 95% CI = 95% confidence interval of the difference, FEV_1_ = Forced expiratory volume in 1 s, FVC = Forced vital capacity, FEV_1_/FVC = Forced expiratory volume in 1 s/forced vital capacity ratio).

**Table 5:** The results of the two-way mixed-model repeated-measures ANOVA for comparing the SBP, DBP, the 3-min step test, and VO_2_max variables among pretest and post-test between each group.

Variables	CON Group (*n* = 9)		MOA Group (*n* = 9)		*p*-Value vs. Time × Group ( $n_{p}^{2}$ )			Between-Group MD (at Week 8) [95% CI]
	Pretest	Post-Test	Pretest	Post-Test	Time	Group	Time × Group	
SBP (mmHg)	117.00 ± 12.36	118.22 ± 8.41	118.90 ± 8.92	107.44 ± 7.35 *	0.036 (0.25)	0.266 (0.08)	0.012 (0.34)	10.78 [2.89, 18.67]
*p*-value vs. group ( $n_{p}^{2}$ )			0.715 (0.01)	0.011 (0.34)				
DBP (mmHg)	69.78 ± 3.83	69.22 ± 4.58	70.11 ± 5.58	69.22 ± 6.69	0.543 (0.02)	0.941 (0.00)	0.888 (0.00)	0.00 [−5.73, 5.73]
*p*-value vs. group ( $n_{p}^{2}$ )			0.575 (0.00)	0.017 (0.00)				
3-min step test (step)	82.33 ± 16.74	77.33 ± 15.89	82.33 ± 13.52	101.00 ± 22.66 *	0.025 (0.28)	0.148 (0.13)	0.001 (0.53)	−23.67 [−43.23, −4.11]
*p*-value vs. group ( $n_{p}^{2}$ )			1.000 (0.00)	0.021 (0.29)				
Submaximal HR (bpm)	148.78 ± 16.74	150.56 ± 6.71	145.56 ± 8.89	137.44 ± 9.90 *	0.002 (13.47)	0.052 (4.42)	<0.001 (32.83)	13.11 [4.66, 21.56]
*p*-value vs. group ( $n_{p}^{2}$ )			0.429 (0.81)	0.005 (3.29)				
VO_2_max (mL·kg^−1^·min^−1^)	53	52	56	58 *	0.887 (0.01)	0.098 (0.16)	0.015 (0.32)	−5.21 [−0.40, −10.03]
*p*-value vs. group ( $n_{p}^{2}$ )			0.228 (0.09)	0.039 (0.24)				

Data are presented as means ± SD. * *p* < 0.05, vs. pretest within the same group. (MD = the mean difference between the MOA and CON groups at post-test, 95% CI = 95% confidence interval of the difference, SBP = Systolic blood pressure, DBP = Diastolic blood pressure, Submaximal HR= Submaximal heart rate, VO_2_max = the maximum rate of oxygen consumption).

## Data Availability

The data that support the findings of this study are available from the first author upon reasonable request.

## Supplementary Materials

The following supporting information can be downloaded at: https://www.mdpi.com/1660-4601/23/5/611/s1, Figure S1: Longitudinal changes and individual responses in HR, RPE, and SpO_2_ at baseline, week 4, and week 8.; Figure S2: Longitudinal changes and individual responses in RSS variables at baseline, week 4, and week 8.; Figure S3: Longitudinal changes and individual responses in PNIF and FeNO at baseline, week 4, and week 8.; Figure S4: Longitudinal changes and individual responses in FEV_1_, FVC, and FEV_1_/FVC following an 8-week intervention.; Figure S5: Longitudinal changes and individual responses in SBP, DBP, the 3-minute step test, Submaximal HR, and VO_2_max following an 8-week intervention.; File S1: CONSORT_2025_editable_checklist [37].

## References

[1] Storms W. (2008). Allergic rhinitis-induced nasal congestion: Its impact on sleep quality. Prim. Care Respir. J..

[2] Park J., Park J.H., Park J., Choi J., Kim T.H. (2020). Association between allergic rhinitis and regular physical activity in adults: A nationwide cross-sectional study. Int. J. Environ. Res. Public Health.

[3] Eksi N., Batur Calis Z.A., Seyhun N., Ozkarafakili A., Coskun B.U. (2021). Evaluation of exercise-induced bronchoconstriction and rhinitis in adolescent elite swimmers. North. Clin. Istanb..

[4] Luo J.-Y., Chen H.-A., Ma J., Xiao Y.-X., Yao J.-J., Liang J.-M., Du Y.-S., Wang F., Sun B.-Q. (2021). Clinical application of fractional exhaled nitric oxide and nasal nitric oxide levels for the assess eosinophilic inflammation of allergic rhinitis among children. Transl. Pediatr..

[5] Ventura M.T., Scichilone N., Paganelli R., Minciullo P.L., Patella V., Bonini M., Passalacqua G., Lombardi C., Simioni L., Ridolo E. (2017). Allergic diseases in the elderly: Biological characteristics and main immunological and non-immunological mechanisms. Clin. Mol. Allergy.

[6] Jiang Y., Yabluchanskiy A., Deng J., Amil F.A., Po S.S., Dasari T.W. (2022). The role of age-associated autonomic dysfunction in inflammation and endothelial dysfunction. Geroscience.

[7] Nieman D.C., Wentz L.M. (2019). The compelling link between physical activity and the body’s defense system. J. Sport Health Sci..

[8] Campbell J.P., Turner J.E. (2018). Debunking the myth of exercise-induced immune suppression: Redefining the impact of exercise on immunological health across the lifespan. Front. Immunol..

[9] Tongtako W., Klaewsongkram J., Jaronsukwimal N., Buranapraditkun S., Mickleborough T.D., Suksom D. (2012). The effect of acute exhaustive and moderate intensity exercises on nasal cytokine secretion and clinical symptoms in allergic rhinitis patients. Asian Pac. J. Allergy Immunol..

[10] Janyacharoen T., Kunbootsri N., Arayawichanon P., Chainansamit S., Sawanyawisuth K. (2015). Responses of six-weeks aquatic exercise on the autonomic nervous system, peak nasal inspiratory flow and lung functions in young adults with allergic rhinitis. Iran. J. Allergy Asthma Immunol..

[11] Sano H. (1992). Influence of environmental temperature (cold exposure) on nasal resistance. Nippon. Jibiinkoka Gakkai Kaiho.

[12] Kerdkaew K., Tongtako W. (2024). Acute Effects of Exercise at Different Temperatures on Clinical Symptoms and Nasal Blood Flow in Patient with Allergic Rhinitis: A Randomized Crossover Trial. Int. J. Exerc. Sci..

[13] Graham B.L., Steenbruggen I., Miller M.R., Barjaktarevic I.Z., Cooper B.G., Hall G.L., Hallstrand T.S., Helgeson K.C., Culham A.B., Swanney M.P. (2019). Standardization of Spirometry 2019 Update. An Official American Thoracic Society and European Respiratory Society Technical Statement. Am. J. Respir. Crit. Care Med..

[14] Bousquet J., Van Cauwenberge P., Khaltaev N. (2001). Allergic rhinitis and its impact on asthma. J. Allergy Clin. Immunol..

[15] Pfaar O., Demoly P., Gerth van Wijk R., Bonini S., Bousquet J., Canonica G.W., Casale T.B., Custovic A., Durham S.R., Kleine-Tebbe J. (2014). Recommendations for the standardization of clinical outcomes used in allergen immunotherapy trials for allergic rhinoconjunctivitis: An EAACI Position Paper. Allergy.

[16] Culver B.H., Graham B.L., Coates A.L., Wanger J., Berry C.E., Clarke P.K., Hallstrand T.S., Hankinson J.L., Kaminsky D.A., MacIntyre N.R. (2017). Recommendations for a standardized pulmonary function report. An official American Thoracic Society technical statement. Am. J. Respir. Crit. Care Med..

[17] Dejsomritrutai W., Nana A., Maranetra K.N., Chuaychoo B., Maneechotesuwan K., Wongsurakiat P., Whangrua P., Naruman C. (2000). Reference spirometric values for healthy lifetime nonsmokers in Thailand. J. Med. Assoc. Thail..

[18] Starling-Schwanz R., Peake H.L., Salome C.M., Toelle B.G., Ng K.W., Marks G.B., Lean M.L., Rimmer S.J. (2005). Repeatability of peak nasal inspiratory flow measurements and utility for assessing the severity of rhinitis. Allergy.

[19] Alving K., Anolik R., Crater G., LaForce C.F., Rickard K. (2017). Validation of a New Portable Exhaled Nitric Oxide Analyzer, NIOX VERO^®^: Randomized Studies in Asthma. Pulm. Ther..

[20] Van Kieu N.T., Jung S.J., Shin S.W., Jung H.W., Jung E.S., Won Y.H., Chung H.W., Lee B.C., Chae S.W. (2020). The validity of the YMCA 3-minute step test for estimating maximal oxygen uptake in healthy Korean and Vietnamese adults. J. Lifestyle Med..

[21] Tongtako W., Klaewsongkram J., Mickleborough T.D., Suksom D. (2018). Effects of aerobic exercise and vitamin C supplementation on rhinitis symptoms in allergic rhinitis patients. Asian Pac. J. Allergy Immunol..

[22] Prossegger J., Huber D., Grafetstätter C., Pichler C., Braunschmid H., Weisböck-Erdheim R., Hartl A. (2019). Winter exercise reduces allergic airway inflammation: A randomized controlled study. Int. J. Environ. Res. Public Health.

[23] Baraniuk J.N., Merck S.J. (2008). Nasal reflexes: Implications for exercise, breathing, and sex. Curr. Allergy Asthma Rep..

[24] Marioni G., Ottaviano G., Staffieri A., Zaccaria M., Lund V., Tognazza E., Coles S., Pavan P., Brugin E., Ermolao A. (2010). Nasal functional modifications after physical exercise: Olfactory threshold and peak nasal inspiratory flow. Rhinology.

[25] Antosova M., Bencova A., Mokra D., Plevkova J., Pepucha L., Buday T. (2020). Exhaled and nasal nitric oxide–impact for allergic rhinitis. Physiol. Res..

[26] Eichenberger P.A., Diener S.N., Kofmehl R., Spengler C.M. (2013). Effects of exercise training on airway hyperreactivity in asthma: A systematic review and meta-analysis. Sports Med..

[27] Hansen E.S.H., Hostrup M., Rasmusen H.K., Hellsten Y., Backer V. (2021). Effect of aerobic exercise training on asthma control in postmenopausal women (the ATOM-study): Protocol for an outcome assessor, randomised controlled trial. BMJ Open.

[28] Zhou L., Xu H. (2023). Feasibility of exercise therapy for children with asthma: A meta-analysis. Front. Cell Dev. Biol..

[29] Krišto B., Maglica M., Petro M., Vidović I., Vukoja A., Hrkać A., Perković R. (2025). Impact of Structured Aerobic Exercise on Symptom Burden and Lung Function in Adults with Asthma: A Prospective Cohort Study. J. Clin. Pract. Res..

[30] Riebe D., Ehrman J.K., Liguori G., Magal M. (2018). ACSM’s Guidelines for Exercise Testing and Prescription.

[31] Nindorera F., Leveque C., Meyer E., Costantino B., Theunissen S. (2025). Optimizing physical fitness in chronic stroke patients: The impact of exercise training modality and dosage on maximal and sub-maximal fitness–a systematic review and meta-analysis. J. Rehabil. Med..

[32] Grant S., Corbett K., Amjad A.M., Wilson J., Aitchison T. (1995). A comparison of methods of predicting maximum oxygen uptake. Br. J. Sports Med..

[33] Noonan V., Dean E. (2000). Submaximal exercise testing: Clinical application and interpretation. Phys. Ther..

[34] Jung W.S., Kim S.W., Park H.Y., Kim J., Lim K. (2021). Effects of acute exposure to thermal stress on cardiorespiratory function, skeletal muscle oxygenation, and exercise performance in healthy males. Int. J. Environ. Res. Public Health.

[35] Silva R.P., Barros C.L., Mendes T.T., Garcia E.S., Valenti V.E., de Abreu L.C., Garner D.M., Espindola F.S., Penha-Silva N. (2019). The influence of a hot environment on physiological stress responses in exercise until exhaustion. PLoS ONE.

[36] Pongkanpai B., Klaewsongkram J., Mickleborough T.D., Tongtako W. (2024). Assessing the Immediate Impact of High-Intensity Interval Exercise on Symptoms and Fractional Exhaled Nitric Oxide Levels. Phys. Act. Health.

[37] Hopewell S., Chan A.W., Collins G.S., Hróbjartsson A., Moher D., Schulz K.F., Tunn R., Aggarwal R., Berkwits M., Berlin J.A. (2025). CONSORT 2025 Statement: Updated guideline for reporting randomised trials. BMJ.

