# Supplementary figures and images for: Effects of Moderate-Intensity Aerobic Exercise on Clinical Symptoms and Physiological Outcomes in Young Adults with Persistent Allergic Rhinitis: A Randomized Controlled Trial
Source: Int J Environ Res Public Health. 2026 May 5;23(5):611. doi: 10.3390/ijerph23050611 (PMC13207304; doi:10.3390/ijerph23050611)

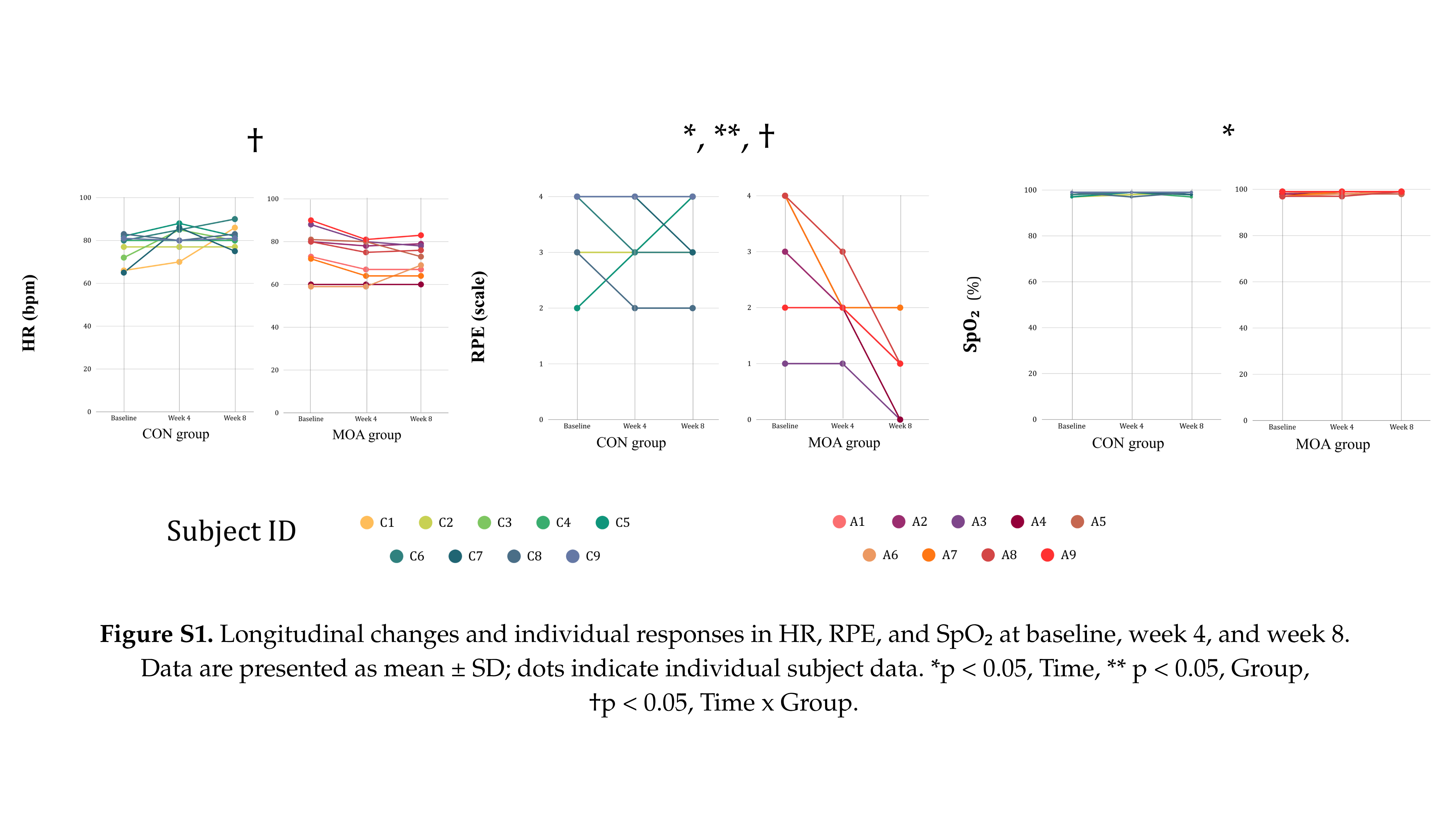

Supplement: Supplementary file 1 [file ijerph-23-00611-s001.zip › Figure S1.png]

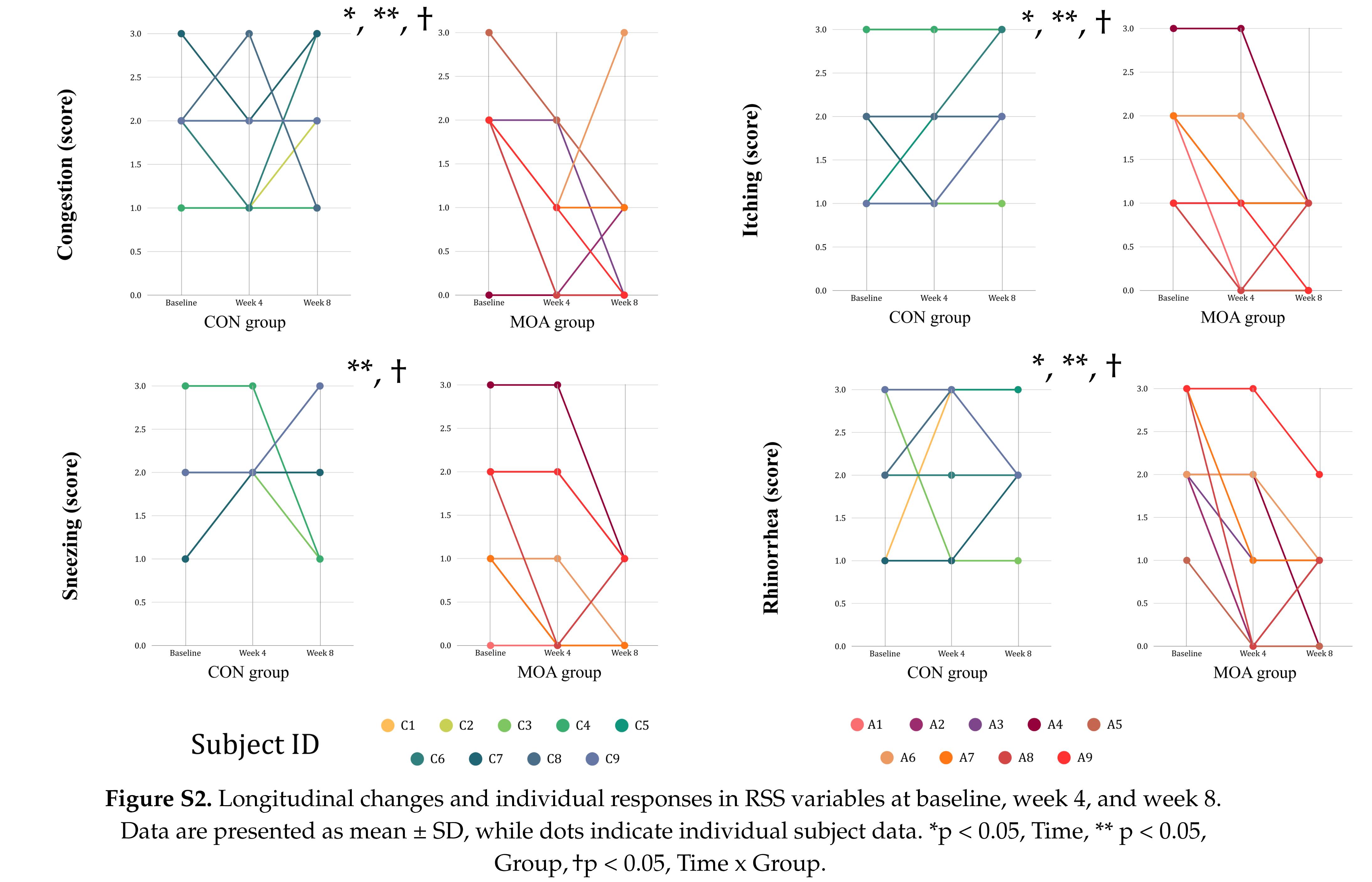

Supplement: Supplementary file 1 [file ijerph-23-00611-s001.zip › Figure S2.png]

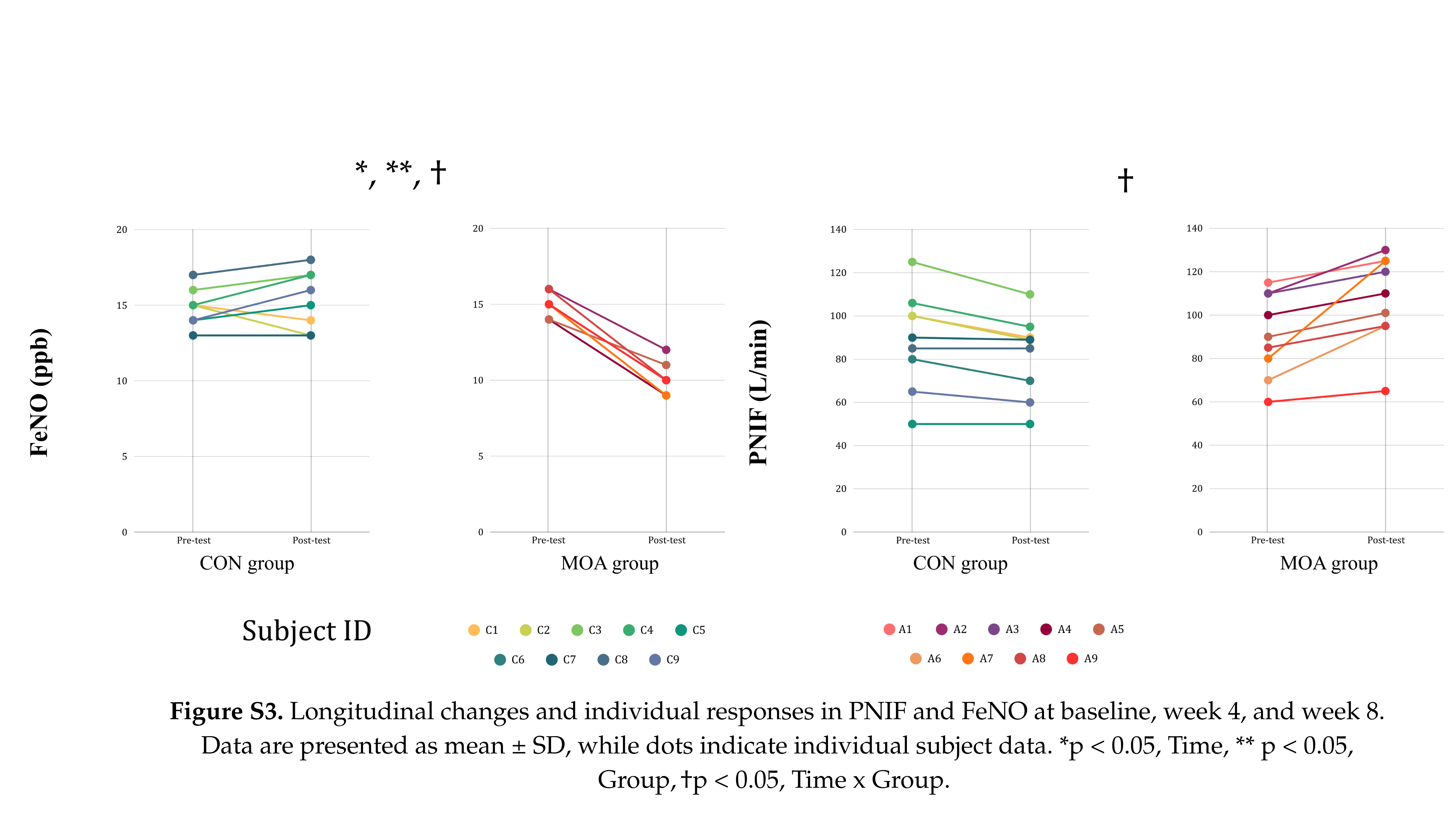

Supplement: Supplementary file 1 [file ijerph-23-00611-s001.zip › Figure S3.png]

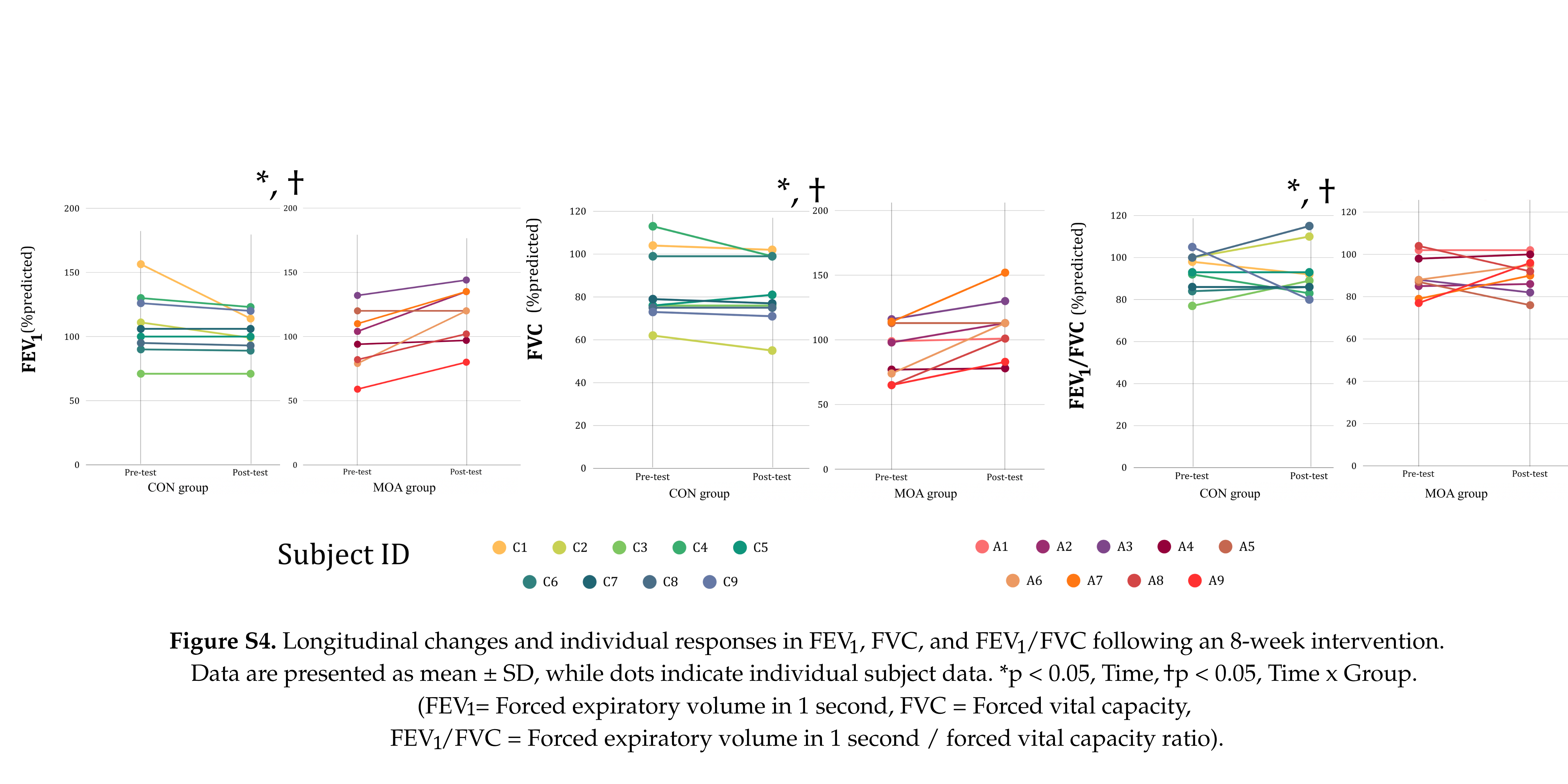

Supplement: Supplementary file 1 [file ijerph-23-00611-s001.zip › Figure S4.png]

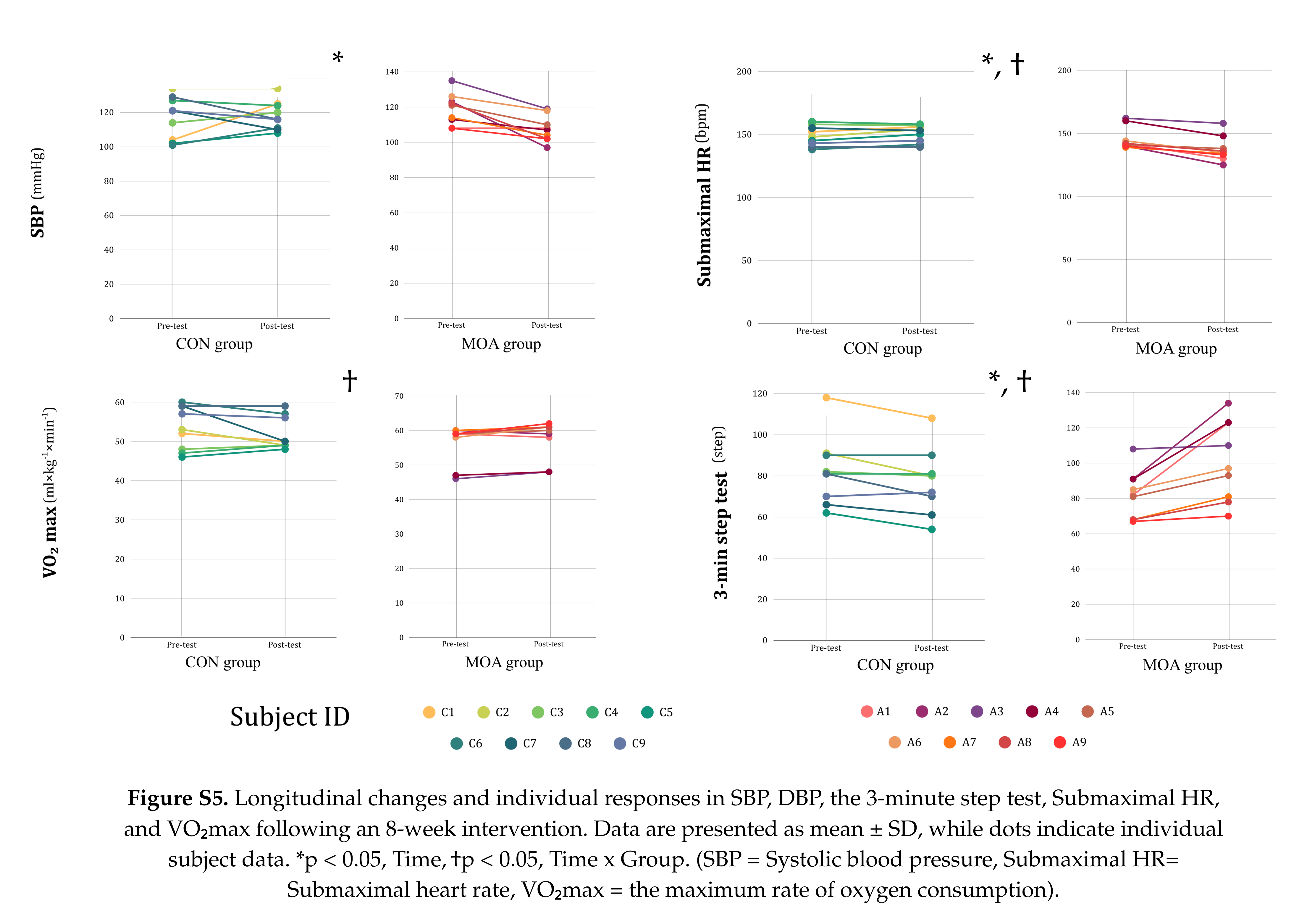

Supplement: Supplementary file 1 [file ijerph-23-00611-s001.zip › Figure S5.png]
